# Genetic Architecture of Parallel Pelvic Reduction in Ninespine Sticklebacks

**DOI:** 10.1534/g3.113.007237

**Published:** 2013-10-01

**Authors:** Takahito Shikano, Veronika N. Laine, Gábor Herczeg, Johanna Vilkki, Juha Merilä

**Affiliations:** * Ecological Genetics Research Unit, Department of Biosciences, University of Helsinki, FI-00014, Helsinki, Finland; † Division of Genetics and Physiology, Department of Biology, University of Turku, FI-20014, Turku, Finland; ‡ MTT Agrifood Research Finland, FI-31600, Jokioinen, Finland

**Keywords:** chromosomal rearrangement, genetic convergence, linkage map, parallel evolution, QTL

## Abstract

Teleost fish genomes are known to be evolving faster than those of other vertebrate taxa. Thus, fish are suited to address the extent to which the same *vs.* different genes are responsible for similar phenotypic changes in rapidly evolving genomes of evolutionary independent lineages. To gain insights into the genetic basis and evolutionary processes behind parallel phenotypic changes within and between species, we identified the genomic regions involved in pelvic reduction in Northern European ninespine sticklebacks (*Pungitius pungitius*) and compared them to those of North American ninespine and threespine sticklebacks (*Gasterosteus aculeatus*). To this end, we conducted quantitative trait locus (QTL) mapping using 283 F_2_ progeny from an interpopulation cross. Phenotypic analyses indicated that pelvic reduction is a recessive trait and is inherited in a simple Mendelian fashion. Significant QTL for pelvic spine and girdle lengths were identified in the region of the *Pituitary homeobox transcription factor 1* (*Pitx1*) gene, also responsible for pelvic reduction in threespine sticklebacks. The fact that no QTL was observed in the region identified in the mapping study of North American ninespine sticklebacks suggests that an alternative QTL for pelvic reduction has emerged in this species within the past 1.6 million years after the split between Northern European and North American populations. In general, our study provides empirical support for the view that alternative genetic mechanisms that lead to similar phenotypes can evolve over short evolutionary time scales.

Understanding the genetic basis of the evolution and diversity of phenotypic traits is a central topic in evolutionary biology. Similar phenotypes often evolve independently across multiple populations or closely related species facing similar environmental selection pressures (Barrett and Schluter 2008; Nadeau and Jiggins 2010). Such parallel phenotypic changes have been observed in a wide variety of organisms (Jones *et al.* 1992; Losos *et al.* 1998; Huey *et al.* 2000; Colosimo *et al.* 2005; Rogers and Bernatchez 2007), strongly suggesting that the observed phenotypic parallelism is shaped by natural selection (Endler 1986; Schluter 2000). Parallel phenotypic evolution can be based on utilization of the same or different genetic mechanisms to acquire the same phenotypes (Arendt and Reznick 2008; Elmer and Meyer 2011). Although several empirical studies have provided examples in which similar phenotypes are produced by the repeated selection of the same pool of standing variation (Barrett and Schluter 2008; Nadeau and Jiggins 2010), the relative importance of standing variation and new mutations in parallel phenotypic evolution is as yet poorly understood (Elmer and Meyer 2011; Conte *et al.* 2012).

Comparative genome mapping provides a framework to assess the similarities and dissimilarities in the genetic bases of phenotypic evolution both at intraspecific and interspecific levels, and also offers an opportunity to gain insights into the evolutionary history of genomes. Recent studies have discovered that the rate of genome evolution differs among taxonomic groups (Kohn *et al.* 2006; Ferguson-Smith and Trifonov 2007; Huttley *et al.* 2007; Hufton *et al.* 2008). For instance, teleost fish genomes exhibit a higher incidence of chromosomal rearrangements and a faster evolution of protein coding sequences than other vertebrate taxa, implying that their genomes are evolving rapidly (Jaillon *et al.* 2004; Naruse *et al.* 2004; Woods *et al.* 2005; Sémon and Wolfe 2007; Hufton *et al.* 2008). Thus, as seen in frequent turnover and rearrangements of fish sex chromosomes (Devlin and Nagahama 2002; Mank and Avise 2009; Cioffi *et al.* 2012; Kitano and Peichel 2012), fish genomes are considered plastic. The rapid evolution of fish genomes is associated with the high morphological, ecological, and physiological diversity in this taxon (Ravi and Venkatesh 2008), and parallel phenotypic evolution has been observed in several fish species (Bell and Foster 1994; Reznick *et al.* 1996; Landry *et al.* 2007; Jeffery 2009; Chan *et al.* 2010). Therefore, teleost fish are well-suited for studying the evolutionary dynamics and processes of genome rearrangements, as well as the genetic basis of parallel phenotypic evolution. In particular, they are useful to address the extent to which the same genes can be responsible for similar phenotypic changes in rapidly evolving genomes.

Stickleback fishes have served as important model organisms in evolutionary biology (Wootton 1976; Bell and Foster 1994; Kingsley and Peichel 2007; Östlund-Nilsson and Mayer 2007; Merilä 2013). In particular, threespine (*Gasterosteus aculeatus*) and ninespine sticklebacks (*Pungitius pungitius*) exhibit diverse phenotypic and ecological characteristics across their global distributions (Bell and Foster 1994; Merilä 2013). Although these species diverged from a common ancestor approximately 13 million years ago (Bell *et al.* 2009), they share similar morphological features, including bony lateral plates and pelvic apparatus. Thus, they are well-suited for studying the genetic basis of phenotypic evolution at both intraspecific and interspecific levels. Parallel phenotypic evolution has been widely observed and extensively studied in threespine sticklebacks (Bell 1976; Bell and Foster 1994; Walker and Bell 2000). Specifically, although ancestral marine threespine sticklebacks have a full row of lateral armor plates, long pelvic spines, and well-developed pelvic girdles, all of these traits have become reduced or even lost in independently and repeatedly colonized freshwater populations in which predation pressure by piscine predators is reduced. Genetic analyses have demonstrated that the repeated reduction in the number of lateral bony plates is governed by the *Ectodysplasin* (*Eda*) gene in globally distributed populations (Colosimo *et al.* 2004, 2005; Cresko *et al.* 2004). Similarly, the *Pituitary homeobox transcription factor 1* (*Pitx1*) gene has been shown to be responsible for pelvic reduction in both Pacific and Atlantic populations of threespine sticklebacks (Cole *et al.* 2003; Cresko *et al.* 2004; Shapiro *et al.* 2004; Coyle *et al.* 2007; Chan *et al.* 2010). Although fewer genetic studies have been conducted using ninespine sticklebacks, phenotypic analyses of F_1_ hybrids between North American ninespine and threespine sticklebacks suggested that pelvic reduction is controlled by the same gene in these species (Shapiro *et al.* 2006). Nevertheless, a quantitative trait locus (QTL) mapping study of North American ninespine sticklebacks indicated that a major genomic region influencing pelvic reduction is distinct from the *Pitx1* locus (Shapiro *et al.* 2009). Thus, genetic variation for pelvic reduction is likely to exist at multiple loci in this species. Phylogenetic analyses have uncovered the presence of several distinct lineages in ninespine sticklebacks across the Northern Hemisphere (Haglund *et al.* 1992a; Takahashi and Goto 2001; Aldenhoven *et al.* 2010; Shikano *et al.* 2010a; Teacher *et al.* 2011). Although threespine sticklebacks have undergone rapid morphological transitions after freshwater colonization, mainly after the last ice age (Bell and Foster 1994), morphological diversification might have occurred through different genetic changes in distinct lineages—and in different time scales—in ninespine sticklebacks because of their more heterogeneous evolutionary history. Therefore, comparative analyses of these two stickleback species can provide important insight into the evolutionary processes and genetic underpinnings that have led to similar phenotypes.

The main aim of this study was to improve our understanding of the genetic basis of parallel pelvic reduction in ninespine sticklebacks and to ask whether this reduction is likely to have occurred through similar or different genetic mechanisms in different lineages. In particular, we were interested in exploring the origin and evolution of genetic variation for pelvic reduction in this species. To this end, we identified the genomic regions involved in pelvic reduction in Northern European ninespine sticklebacks—with the aid of QTL mapping with 283 F_2_ segregating progeny of the cross between populations with and without pelvic spines—and compared them to those of North American ninespine and threespine sticklebacks (Cole *et al.* 2003; Cresko *et al.* 2004; Shapiro *et al.* 2004, 2006, 2009; Coyle *et al.* 2007; Chan *et al.* 2010). In addition, intraspecific and interspecific comparative genomic analyses were conducted to assess possible chromosomal rearrangements by comparing our linkage map with that of North American ninespine sticklebacks (Shapiro *et al.* 2009) and the threespine stickleback genome (Jones *et al.* 2012). Given that the stickleback sex chromosomes are known to evolve in rapid pace (Ross *et al.* 2009), we also mapped the sex-determining locus in the European lineage of ninespine sticklebacks to see whether it corresponds to that found in the North American lineage of this species.

## Materials and Methods

### Fish

The grandparental fish (F_0_) were collected from the Baltic Sea (Helsinki, Finland; 60°13′N, 25°11′E) and a pond (Rytilampi, Finland; 66°23′N, 29°19′E) in northeastern Finland in 2006. Pelvic reduction was observed in the pond population, whereas no pelvic reduction was found in the marine population (Herczeg *et al.* 2010). A female from the marine population was artificially crossed with a male from the pond population in July 2006, and the F_1_ offspring were reared in an aquarium at approximately 15°. They were fed brine shrimp (*Artemia* sp.) nauplii in larval and juvenile stages, and, later, frozen bloodworms (*Chironomidae* sp.). After an artificial hibernation at 6° without light, fish were maintained at 17° under permanent light to facilitate reproduction. One female and one male were randomly chosen and mated in an aquarium. Seven successive clutches were obtained in September and October 2008. These clutches were reared in 1.4-liter tanks in a zebrafish rack system equipped with physical, biological, and UV filters (Aquaneering Inc., San Diego, CA). The F_2_ offspring were placed individually in 1.4-liter tanks in the zebrafish rack systems 6 d after hatching. White plastic sheets were placed between the tanks to block visibility between the fish. The fish were reared at 17° under the 14-hr light and 10-hr dark photoperiod and fed twice per day. Food was, at first, brine shrimp nauplii. After 4 wk, it was changed to brine shrimp nauplii and frozen *Cyclops*. After another 4 wk, it was changed to frozen *Cyclops* and bloodworms. Finally, after another 4 wk, it was changed to frozen bloodworms. At 187 days posthatching, the fish were anesthetized with MS-222 (tricaine methanesulfonate) and photographed with a digital camera. The specimens were fixed in 4% formalin for morphological measurements. Fin clips were preserved in ethanol for DNA analyses. In total, 283 fish (56, 44, 38, 37, 40, 31, and 27 from seven clutches) were used for analyses. The experiments were conducted under the license from the Finnish National Animal Experiment Board (#STH379A).

### Morphological measurements

The fish were stained with Alizarin Red S, following the study of Pritchard and Schluter (2001). Pelvic spine and girdle lengths were measured with a digital caliper to the nearest 0.01 mm. Both left and right pelvic girdles and spines were measured twice by the same person, and the averaged values were used for analyses. As a size proxy, we calculated centroid size, which is the square root of the summed squared distances from the landmarks to the centroid (Bookstein 1991). We used the same landmarks on the digital photographs as outlined by Herczeg *et al.* (2010), and we calculated centroid size using tpsRelw 1.46 (Rohlf 2006). Gender was determined by checking the gonads.

### DNA extraction and genotyping

Total genomic DNA was extracted from ethanol-preserved fin clips using a silica fine-based purification method (Elphinstone *et al.* 2003) after proteinase K digestion. All the F_2_ offspring (*N* = 283) and their parents and grandparents (*N* = 4) were genotyped for 235 microsatellite markers (Largiadèr *et al.* 1999; Peichel *et al.* 2001; Heckel *et al.* 2002; Colosimo *et al.* 2004; Miller *et al.* 2007; Mäkinen *et al.* 2008; Shapiro *et al.* 2009; Shikano *et al.* 2010b, 2011; Shimada *et al.* 2011; Laine *et al.* 2012; Supporting Information, Table S1). Out of the 235 markers, 46 were developed for specific genes with known biological functions in fish (Shapiro *et al.* 2009; Shikano *et al.* 2010b; Shimada *et al.* 2011; Laine *et al.* 2012). Polymerase chain reactions (PCRs) for all markers except Ppbig were performed in a 10-μl volume containing 1× Qiagen Multiplex PCR Master Mix (Qiagen), 0.5× Q-Solution, 2 pmol of each primer, and 10–20 ng of template DNA. One of each primer pair was labeled with FAM, HEX, or TET fluorescent dye. PCR cycling started with an initial activation step at 95° for 15 min, followed by 30 cycles of 94° for 30 sec, 55° for 90 sec, and 72° for 60 sec, and completed with a final extension at 60° for 5 min. PCRs for Ppbig markers were conducted according to the methods of Laine *et al.* (2012). All PCR products were diluted 1:500 with Milli-Q water and genotyped using a MegaBACE 1000 automated sequencer (Amersham Biosciences) with ET-ROX 400 size standard (Amersham Biosciences). Alleles were scored using Fragment Profiler 1.2 program (Amersham Biosciences) and edited by eye. To ensure consistency in genotyping, all alleles were read by the same person.

### Linkage map

A linkage map was constructed using improved CRI-MAP 2.5 (Green *et al.* 1990). The logarithm of the odds (LOD) scores for all pairs of markers were obtained using the TWOPOINT option. LOD score threshold of 3.0 was used as a significant criterion for linkage. The linkage map of North American ninespine sticklebacks (Shapiro *et al.* 2009) and the threespine stickleback genome sequence (Ensembl, database v. 66.1) were used as a reference for the initial linkage group (LG) building. For each LG, the best order of the markers was determined using the BUILD option by beginning with the most informative marker pair. Markers that could not be fitted straight with BUILD and with LOD score ≥4.0 were fitted manually. The FLIPS option (*N* = 3–5) was used to evaluate the statistical significance of the obtained order. After the best order was determined within each LG, double recombination events were detected using the CHROMPIC option. Individuals with more than four recombinations were removed and a second CRIMAP analysis round was conducted. Out of the 235 markers used for linkage analyses, nine with low polymorphism were discarded because of low LOD scores in TWOPOINT. Linkage maps were drawn using MAPCHART 2.2 (Voorrips 2002).

Genomic synteny was investigated by comparing our linkage map with the threespine stickleback genome (Jones *et al.* 2012) and the linkage map of North American ninespine sticklebacks (Shapiro *et al.* 2009). For the markers developed specifically for ninespine sticklebacks, microsatellite flanking sequences were subject to BLASTN searches against the threespine stickleback genome to identify homologous genomic regions. BLAST hits were considered significant at a threshold of E < 10^−5^. Out of 226 informative markers in our linkage map, 110 were used for the linkage map of North American ninespine sticklebacks (Shapiro *et al.* 2009).

### QTL mapping

QTL analyses were conducted with 226 markers, each of which had genotyping success of the F_2_ offspring between 74% and 99%. Sex-averaged linkage map distances were used for QTL mapping. The analyses were performed with GridQTL (Seaton *et al.* 2006) by using the BCF2 portlet and fitting both additive and dominance effects at 1-cM intervals (File S1, File S2, and File S3). Sex was included in the models as a fixed effect, and centroid size was included as a covariate. Experiment-wide and chromosome-wide significance levels of QTL were determined based on 10,000 permutations. QTL was considered significant when the *F*-value was more than the 5% experiment-wide threshold, and was considered suggestive when the *F*-value was more than the 5% chromosome-wide threshold. Confidence intervals were estimated with 10,000 bootstrap iterations. The proportion of the phenotypic variance explained by the QTL was calculated according to the methods of Zhou *et al.* (2006).

## RESULTS

### Linkage and comparative mapping

The genetic linkage map constructed with 226 informative markers consisted of 21 LGs (Figure S1), in accord with chromosome number based on cytogenetic analysis (Ocalewicz *et al.* 2008). The sex-averaged linkage map spanned 1,632.7 cM, with an average intermarker distance of 7.2 cM. The number of loci per LG ranged from 5 to 21, and the size of the LGs spanned from 31.1 to 121.3 cM (Table S2). The female map covered 2178.7 cM and the male map covered 1211.6 cM (Table S2). Out of the 226 markers mapped, 217 had significant similarity to sequences of the threespine stickleback genome (Table S1). All of the 217 markers except one were assigned to specific LGs of the threespine stickleback genome.

**Figure 1:**
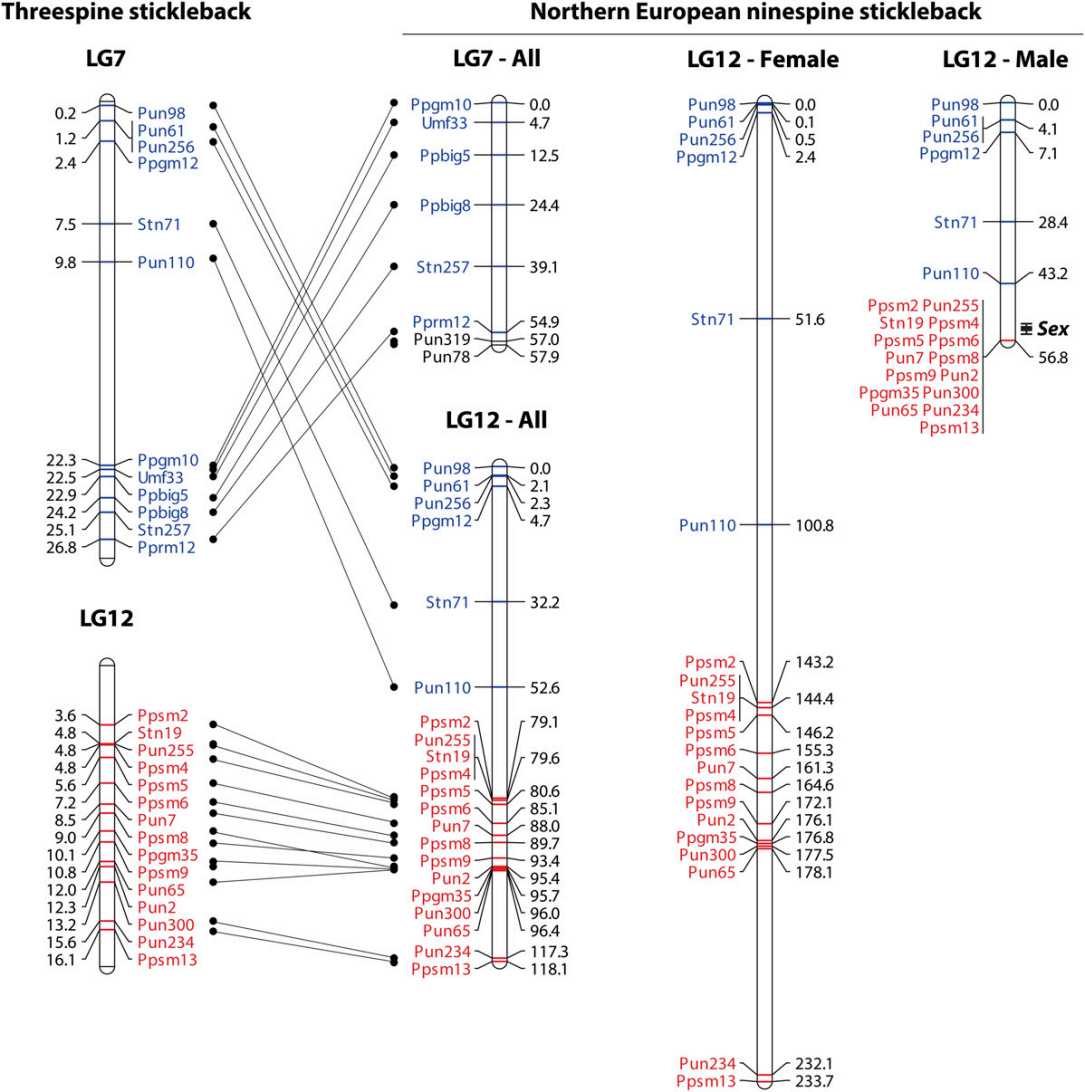
Synteny of LG7 and LG12 between the threespine and Northern European ninespine sticklebacks and sex-specific maps of LG12 in the Northern European ninespine stickleback. Marker positions are given in Mb for the threespine stickleback and in cM for the Northern European ninespine stickleback. Markers belonging to LG7 and LG12 in the threespine stickleback are indicated in blue and red, respectively. Location of the sex-determining locus (*Sex*) is shown in the male map with 95% confidence interval.

In the linkage map, 206 markers were located in LGs in accordance with those of the threespine stickleback genome (Table 1). Likewise, in a comparison with the linkage map of North American ninespine sticklebacks (Shapiro *et al.* 2009), 105 out of the 110 markers used in both studies were mapped to the same LGs (Table 1). Although 12 markers were located on LG7 in the threespine stickleback genome, six of them were mapped to LG12, together with a set of 15 markers belonging to LG12 in the threespine stickleback genome (Figure 1). In the 21 markers mapped to LG12, pairwise LOD scores between the markers belonging to threespine stickleback LG7 and LG12 were significant (≥3.0) in 30 out of 90 combinations. However, the six markers belonging to threespine stickleback LG7 showed no significant LOD scores in any of 48 pairwise comparisons with the markers mapped to LG7 (Table S3). In addition to the interchromosomal discordance, the marker order within each LG was consistently inverted between the threespine stickleback genome and the linkage map of Northern European ninespine sticklebacks in partial regions of several chromosomes, including LG1, LG4, LG5, LG8, LG9, LG11, and LG13 (Figure S2).

**Table 1:** Synteny between the Northern European ninespine stickleback and threespine stickleback genome and between the Northern European and North American ninespine sticklebacks

Species	Northern European Ninespine Stickleback																				
LG	LG1	LG2	LG3	LG4	LG5	LG6	LG7	LG8	LG9	LG10	LG11	LG12	LG13	LG14	LG15	LG16	LG17	LG18	LG19	LG20	LG21
Threespine stickleback																					
LG1	15	—	—	—	—	—	—	—	—	—	—	—	—	—	—	—	—	—	—	—	—
LG2	—	8	—	—	—	—	—	—	—	—	—	—	—	—	—	—	—	—	—	—	—
LG3	—	—	9	—	—	—	—	—	—	—	—	—	—	—	—	—	—	—	—	—	—
LG4	—	—	—	16	—	—	—	—	—	—	—	—	—	—	—	—	—	—	1	—	—
LG5	—	—	1	—	9	—	—	—	—	—	—	—	—	—	—	—	—	—	—	—	—
LG6	—	—	—	—	—	6	—	—	—	—	—	—	—	—	—	—	—	—	—	—	—
LG7	—	—	—	—	—	—	6	—	—	—	—	6	—	—	—	—	—	—	—	—	—
LG8	—	—	—	—	—	—	—	9	—	—	—	—	—	—	—	—	—	—	—	—	—
LG9	—	—	—	—	—	1	—	—	10	—	—	—	—	—	—	—	—	—	—	—	—
LG10	—	—	—	—	—	—	—	—	—	7	—	—	—	—	—	—	—	—	—	—	—
LG11	—	—	—	—	—	—	—	—	—	—	18	—	—	—	—	—	—	—	—	—	—
LG12	—	—	—	—	—	—	—	—	—	—	—	15	—	—	—	—	—	—	—	—	—
LG13	—	—	—	—	—	—	—	—	—	—	—	—	16	—	—	—	—	—	1	—	—
LG14	—	—	—	—	—	—	—	—	—	—	—	—	—	4	—	—	—	—	—	—	—
LG15	—	—	—	—	—	—	—	—	—	—	—	—	—	—	10	—	—	—	—	—	—
LG16	—	—	—	—	—	—	—	—	—	—	—	—	—	—	—	11	—	—	—	—	—
LG17	—	—	—	—	—	—	—	—	—	—	—	—	—	—	—	—	7	—	—	—	—
LG18	—	—	—	—	—	—	—	—	—	—	—	—	—	—	—	—	—	6	—	—	—
LG19	—	—	—	—	—	—	—	—	—	—	—	—	—	—	—	—	—	—	11	—	—
LG20	—	—	—	—	—	—	—	—	—	—	—	—	—	—	—	—	—	—	—	6	—
LG21	—	—	—	—	—	—	—	—	—	—	—	—	—	—	—	—	—	—	—	—	7
Unknown	2	—	—	—	—	—	2	—	1	—	—	—	1	1	—	—	2	1	—	—	—
North American ninespine stickleback																					
LG1A	7	—	—	—	—	—	—	—	—	—	—	—	—	—	—	—	—	—	—	—	—
LG1B	2	—	—	—	—	—	—	—	—	—	—	—	—	—	—	—	—	—	—	—	—
LG2	—	1	—	—	—	—	—	—	—	—	—	—	—	—	—	—	—	—	—	—	—
LG3	—	—	4	—	—	—	—	—	—	—	—	—	—	—	—	—	—	—	—	—	—
LG4	—	—	—	8	—	—	—	—	—	—	—	—	—	—	—	—	—	—	—	—	—
LG5A	—	—	—	—	6	—	—	—	—	—	—	—	—	—	—	—	—	—	—	—	—
LG5B	—	—	—	—	—	—	—	—	—	—	—	—	—	—	—	—	—	—	—	—	—
LG6A	—	—	—	—	—	2	—	—	—	—	—	—	—	—	—	—	—	—	—	—	—
LG6B	—	—	—	—	—	1	—	—	—	—	—	—	—	—	—	—	—	—	—	—	—
LG7A	—	—	—	—	—	—	—	—	—	—	—	5	—	—	—	—	—	—	—	—	—
LG7B	—	—	—	—	—	—	2	—	—	—	—	—	—	—	—	—	—	—	—	—	—
LG8	—	—	—	—	—	—	—	3	—	—	—	—	—	—	—	—	—	—	—	—	—
LG9A	—	—	—	—	—	—	—	—	3	—	—	—	—	—	—	—	—	—	—	—	—
LG9B	—	—	—	—	—	—	—	—	1	—	—	—	—	—	—	—	—	—	—	—	—
LG10	—	—	—	—	—	—	—	—	—	5	—	—	—	—	—	—	—	—	—	—	—
LG11A	—	—	—	—	—	—	—	—	—	—	7	—	—	—	—	—	—	—	—	—	—
LG11B	—	—	—	—	—	—	—	—	—	—	1	—	—	—	—	—	—	—	—	—	—
LG12	—	—	—	—	—	—	—	—	—	—	—	6	—	—	—	—	—	—	—	—	—
LG13	—	—	—	—	—	—	—	—	—	—	—	—	11	—	—	—	—	—	—	—	—
LG14A	—	—	—	—	—	—	—	—	—	—	—	—	—	—	—	—	—	—	—	—	—
LG14B	—	—	—	—	—	—	—	—	—	—	—	—	—	2	—	—	—	—	—	—	—
LG15A	—	—	—	—	—	—	—	—	—	—	—	—	—	—	1	—	—	—	—	—	—
LG15B	—	—	—	—	—	—	—	—	—	—	—	—	—	—	5	—	—	—	—	—	—
LG16	—	—	—	—	—	—	—	—	—	—	—	—	—	—	—	6	—	—	—	—	—
LG17	—	—	—	—	—	—	—	—	—	—	—	—	—	—	—	—	5	—	—	—	—
LG18	—	—	—	—	—	—	—	—	—	—	—	—	—	—	—	—	—	2	—	—	—
LG19	—	—	—	—	—	—	—	—	—	—	—	—	—	—	—	—	—	—	8	—	—
LG20A	—	—	—	—	—	—	—	—	—	—	—	—	—	—	—	—	—	—	—	3	—
LG20B	—	—	—	—	—	—	—	—	—	—	—	—	—	—	—	—	—	—	—	—	—
LG21	—	—	—	—	—	—	—	—	—	—	—	—	—	—	—	—	—	—	—	—	3

The numbers of markers on respective linkage groups are indicated. The data of the North American ninespine stickleback are based on the work of Shapiro *et al.* (2009).

### Mapping of the sex-determining locus

The sex-determining locus was mapped to LG12, with the peak value at 53.2 cM (95% C.I. = 52.0–55.0) in the male map (*F* = 159.1; LOD = 46.1; Figure 1). In this LG, the sex-specific maps of females and males were 233.7 cM and 56.8 cM, respectively, including six markers belonging to threespine stickleback LG7 (Figure 1). In the male meiosis, no recombination was observed between the 15 markers belonging to threespine stickleback LG12 (Figure 1) or between phenotypic sex and male-linked alleles at these loci (Table S4).

### Mapping of pelvic reduction

**Figure 2:**
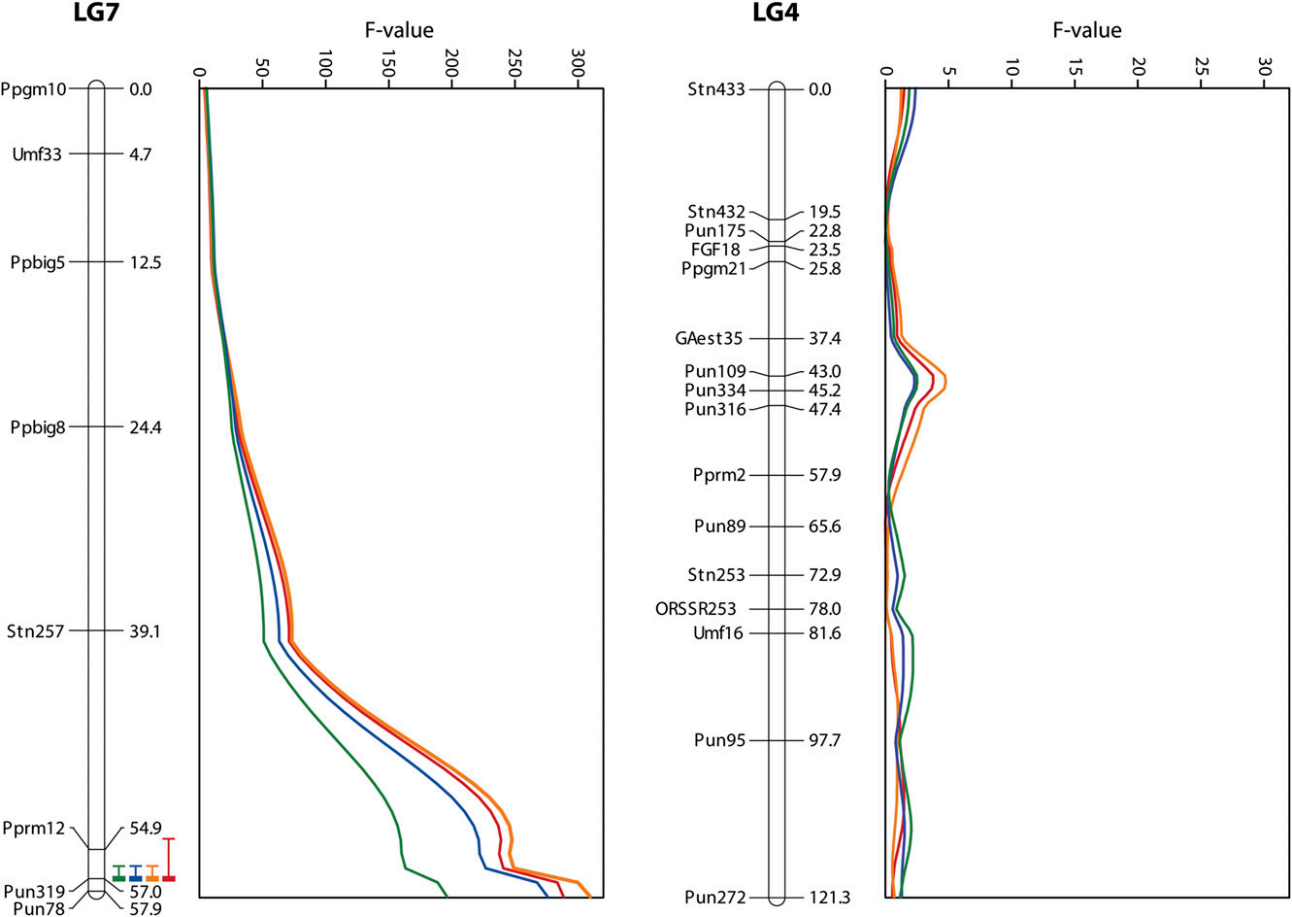
QTL location with 95% confidence interval and *F*-value for left (red and blue) and right (orange and green) pelvic spine (red and orange) and girdle (blue and green) lengths in LG7 and LG4. Marker positions are given in cM.

In the F_2_ progeny of F_1_ hybrids (all F_1_ fish had pelvic spines), pelvic spines were present in 218 individuals and absent in 65 individuals, in agreement with a 3:1 Mendelian ratio (χ^2^ = 0.62; *P* = 0.43). Mapping analyses detected a significant QTL for the lengths of left and right pelvic spines and girdles that exhibited extremely high *F*-values (196.1–309.6) and LOD scores (53.1–70.7) (Figure 2, Table 2). All of these traits were mapped to LG7 at 57 cM, the end of the LG (95% C.I. = 54–57 for left pelvic spine length and 56–57 for other traits) at marker Pun319, which is located within the *Pitx1* gene (Shapiro *et al.* 2009). This QTL region explained 58–69% of phenotypic variance in these traits (Table 2). In the analyses of different genotypes for three different alleles at Pun319, considerably lower mean values of these traits were observed for individuals with two alleles from Rytilampi than for those with other allelic combinations (ANOVA, *F*_3,267_ = 118.3–227.0; *P* < 0.001; Table 2). Individuals with alleles from both Helsinki and Rytilampi tended to show slightly lower mean values as compared to those with the homozygous alleles of Helsinki, exhibiting significant differences in four out of the eight comparisons (Tukey test, *P* < 0.05).

**Table 2:** QTL and phenotypic variation of pelvic spine and girdle lengths

							Phenotypic Mean (± SE) for Each Genotype at Pun319 (mm)			
Trait	LG	Position in cM (C.I.)	*F*	LOD	PVE (%)	Closest Marker	HEL/HEL (*N* = 70)	HEL/RYT1 (*N* = 60)	HEL/RYT2 (*N* = 73)	RYT1/RYT2 (*N* = 68)
Left pelvic spine	LG7	57 (54–57)	288.34	67.80	67	Pun319	3.62 ± 0.09	3.22 ± 0.11	3.41 ± 0.07	0.47 ± 0.13
Right pelvic spine	LG7	57 (56–57)	309.61	70.73	69	Pun319	3.49 ± 0.09	3.23 ± 0.11	3.32 ± 0.07	0.35 ± 0.12
Left pelvic girdle	LG7	57 (56–57)	275.72	65.99	66	Pun319	7.98 ± 0.13	7.44 ± 0.16	7.41 ± 0.09	3.90 ± 0.18
Right pelvic girdle	LG7	57 (56–57)	196.05	53.11	58	Pun319	7.61 ± 0.13	7.14 ± 0.16	7.11 ± 0.08	4.37 ± 0.17

C.I., 95% confidence interval; PVE, proportion of phenotypic variation explained; HEL, allele from Helsinki; RYT, allele from Rytilampi.

No significant or suggestive QTL were detected for these traits in other LGs, including LG4 (Figure 2), in which a strong QTL for these traits was identified in North American ninespine sticklebacks (Shapiro *et al.* 2009).

## Discussion

### Genetic basis of parallel pelvic reduction

Our study uncovered a major genomic region determining pelvic reduction in Northern European ninespine sticklebacks. Based on the phenotypic segregation in the interpopulation cross, it is apparent that the reduction of pelvic spines is recessive and inherited in a simple Mendelian fashion, as observed in threespine sticklebacks (Cresko *et al.* 2004; Shapiro *et al.* 2004; Coyle *et al.* 2007). Both pelvic spine and girdle lengths were mapped to the same region where the *Pitx1* gene is located, explaining large proportions of the variance in these traits. This gene is known to be responsible for pelvic reduction in threespine sticklebacks (Cole *et al.* 2003; Cresko *et al.* 2004; Shapiro *et al.* 2004; Coyle *et al.* 2007; Chan *et al.* 2010). Thus, the *Pitx1* gene is a strong candidate for the determination of pelvic phenotypes in Northern European ninespine sticklebacks.

**Figure 3:**
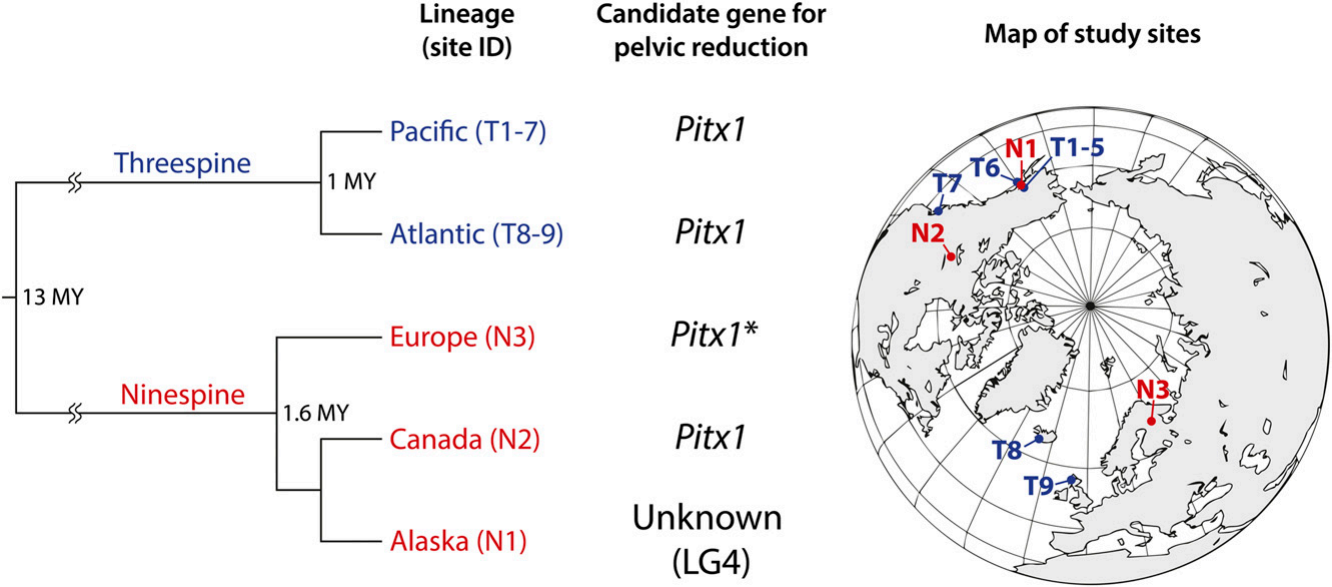
Phylogenetic relationships and candidate genes for pelvic reduction in threespine and ninespine sticklebacks. The phylogeny is based on molecular (Haglund *et al.* 1992b; Ortí *et al.* 1994; Aldenhoven *et al.* 2010; Teacher *et al.* 2011) and fossil data (Bell *et al.* 2009). The candidate genes were determined by the previous (Cole *et al.* 2003; Cresko *et al.* 2004; Shapiro *et al.* 2004, 2006, 2009; Coyle *et al.* 2007; Chan *et al.* 2010) and current* studies.

Shapiro *et al.* (2006) conducted interspecific crosses between threespine and ninespine sticklebacks to test whether the same gene or a different gene is responsible for pelvic reduction in these two species. Based on the phenotypes of parents and their F_1_ hybrid progeny, they suggested that the same genetic mechanism is likely to be involved in pelvic reduction in the two species. However, in a mapping study of ninespine sticklebacks with the F_1_ individuals of a cross between parents lacking pelvic structures from phenotypically monomorphic Canadian and polymorphic Alaskan populations, the major genomic region involved in pelvic reduction was identified on LG4, which was completely distinct from the *Pitx1* locus on LG7 (Shapiro *et al.* 2009). This region explained large proportions (81–87%) of the phenotypic variance of pelvic spine and girdle lengths in their cross, thus exhibiting a strong genetic impact on these traits (Shapiro *et al.* 2009). Given that the *Pitx1* gene appears to be involved in pelvic reduction in the Canadian population (Shapiro *et al.* 2006), it is likely that pelvic reduction is caused by different genes in these populations, implying that genetic variation resulting in similar phenotypes of pelvic structures exists in at least two different loci in North American ninespine sticklebacks. Based on our and previous studies (Shapiro *et al.* 2006, 2009), it appears that although the same genetic mechanism for pelvic reduction might have persisted in threespine and ninespine sticklebacks during 13 million years of divergence (Bell *et al.* 2009), an alternative genetic mechanism for pelvic reduction may have evolved in North American ninespine sticklebacks, possibly within the past 1.6 million years after the divergence from Northern European ninespine sticklebacks (Teacher *et al.* 2011) (Figure 3). Further mapping analyses with more populations from Northern Europe and North America should provide a more refined picture of the evolutionary process of different genetic architectures for pelvic reduction in sticklebacks.

It has been suggested that the likelihood of genetic parallelism underlying similar phenotypic changes decreases with increasing evolutionary distance between taxa (Elmer and Meyer 2011; Conte *et al.* 2012). There are several known cases in which the same genes are responsible for phenotypic parallelism within the same or closely related species (Wood *et al.* 2005; Arendt and Reznick 2008; Nadeau and Jiggins 2010). However, the same gene can underlie similar phenotypes even across disparate taxonomic groups (Arendt and Reznick 2008; Nadeau and Jiggins 2010; Conte *et al.* 2012). The *Melanocortin 1 receptor* (*Mc1r*) gene is a case in point; it is known to control for pigmentation variation in diverse vertebrate taxa, including mammals, birds, and reptiles (Manceau *et al.* 2010). The most probable form of genetic parallelism among distantly related taxa is provided by repeated independent mutations in the same gene rather than shared ancestral genetic variation (Elmer and Meyer 2011). Thus far, empirical studies to infer parallel genetic evolution have been mostly based on candidate gene approaches, which rely on *a priori* hypothesis with respect to coding sequence or expression differences in a particular gene of interest (Wood *et al.* 2005; Arendt and Reznick 2008; Nadeau and Jiggins 2010). Consequently, inferences about different genetic mechanisms can be made solely on the basis of the lack of evidence for genetic parallelism in candidate genes. In contrast, genetic mapping provides powerful means to identify the locations and magnitudes of genomic regions controlling various phenotypic traits on a genome-wide scale. Despite the fact that intraspecific comparative mapping has been rarely performed—partly because of logistical limitations to establish mapping crosses—a few recent studies have begun to identify different genes or genomic regions involved in similar phenotypes within the same species (Gross *et al.* 2009; Thurber *et al.* 2013) as well as between closely related species (Ng *et al.* 2008; Lee *et al.* 2011). Although generalizations about the genetic mechanisms and evolutionary processes that lead to similar phenotypes may as yet be too early (Kopp 2009; Elmer and Meyer 2011), the case of pelvic reduction in ninespine sticklebacks highlights the fact that alternative QTL resulting in similar phenotypes can evolve over a short evolutionary time scale. This emphasizes the importance of both interspecific and intraspecific comparisons in understanding the genetic architectures of parallel phenotypic evolution.

In addition to a major genomic region explaining 81–87% of the variance in pelvic structures, a modifier locus of smaller effect was mapped to LG1 in North American ninespine sticklebacks (Shapiro *et al.* 2009). Similarly, four modifier loci were identified in threespine sticklebacks in which the *Pitx1* locus explained 65% and 47% of the variance in pelvic spine and girdle lengths, respectively (Shapiro *et al.* 2004). Although the *Pitx1* locus explained similar proportions of the variance in these traits in Northern European ninespine sticklebacks as observed in threespine sticklebacks (Shapiro *et al.* 2004), no significant modifier loci were detected in our study. Based on these results, it is likely that the number and location of modifier loci with small effects can differ between populations or species in sticklebacks, possibly because of their different genetic backgrounds. It should be also noted that the possible existence of modifier loci with small effects in the European lineage cannot be excluded because the power of QTL mapping largely depends on the numbers of individuals and loci screened (Hu and Xu 2008; Slate *et al.* 2010). Nevertheless, it is worth noticing that the numbers of progeny (283 *vs.* 120) and loci (226 *vs.* 190) screened in this study were larger than those in the study by Shapiro *et al.* (2009).

Closely related populations are expected to have the same or similar genetic bases of parallel phenotypic changes because of their similar genetic backgrounds and selection trajectories (Wood *et al.* 2005; Barrett and Schluter 2008; Pritchard *et al.* 2010). In threespine sticklebacks, rapid morphological transitions after freshwater colonization have occurred in fish belonging to the Pacific and Atlantic clades, which diverged approximately one million years ago (Haglund *et al.* 1992b; Ortí *et al.* 1994; Higuchi and Goto 1996). Based on genetic analyses with globally distributed populations, it appears that the reductions of lateral plates and pelvic structures have evolved via the same genetic mechanisms in the different lineages, implying that the ancestral marine threespine sticklebacks of these lineages shared the same or similar genetic variation for these traits (Cresko *et al.* 2004; Shapiro *et al.* 2004; Colosimo *et al.* 2005; Coyle *et al.* 2007). However, even though the same major gene is responsible for the reduction of lateral plates in a number of freshwater populations, an exception was found in one Japanese population, which is located in the southernmost area of their distribution range (Colosimo *et al.* 2005). This population is thought to have colonized freshwater approximately 40,000 years ago during the middle Pleistocene period (Watanabe *et al.* 2003), whereas freshwater colonization in other populations has occurred mostly within the past 12,000 years in postglacial times (Bell and Foster 1994). Hence, the different colonization histories might be associated with the occurrence of an alternative genetic mechanism for lateral plate reduction. In contrast to threespine sticklebacks, ninespine sticklebacks comprise several divergent lineages, providing more potential for their independent evolution (Haglund *et al.* 1992a; Takahashi and Goto 2001; Aldenhoven *et al.* 2010; Shikano *et al.* 2010a). Therefore, the opportunity to evolve alternative genetic mechanisms for similar phenotypes may be increased, as found in the case of pelvic reduction of this species. Further interspecific and intraspecific comparative studies with sticklebacks may provide important insights into the genetic underpinnings of parallel phenotypic evolution and the factors underlying the occurrence of different genetic mechanisms that lead to similar phenotypes.

### Chromosomal rearrangements

The comparative mapping revealed that a segment of one autosome corresponding to LG7 in threespine sticklebacks is linked to LG12 in Northern European ninespine sticklebacks. Our results also indicated that none of the loci located on this segmental part shows linkage to the markers mapped to LG7. Hence, the rearrangement of genetic linkage patterns is likely attributable to a chromosomal rearrangement that has occurred after the divergence between threespine and ninespine sticklebacks. Because no linkage was detected between the segmental part and either the remaining region of LG7 or LG12 in the genetic map of North American ninespine sticklebacks (Shapiro *et al.* 2009), it is not certain if the arrangement of linkage patterns has occurred before or after the split between Northern European and North American populations. Our study also identified possible chromosomal inversions in several LGs as compared to the threespine stickleback genome, although potential errors in the genome sequences cannot be ruled out (Ross and Peichel 2008; Natri *et al.* 2013). Further cytogenetic analyses would clarify whether the linkage between LG12 and the segment of LG7 has formed via physical or pseudo linkage, as well as verify the occurrence of intrachromosomal rearrangements in Northern European ninespine sticklebacks.

Although rapid turnover of sex chromosome systems is often observed in fish, including sticklebacks (Devlin and Nagahama 2002; Mank and Avise 2009), the sex-determining gene was mapped to LG12 in Northern European ninespine sticklebacks, as in North American fish (Shapiro *et al.* 2009). In the male meiosis of both North American and Northern European ninespine sticklebacks, no recombination was observed in the chromosomal region corresponding to threespine stickleback LG12. Nevertheless, it is noteworthy that the interchromosomal rearrangement of linkage patterns was found for the sex chromosomes in our study. Cytogenetic analyses have shown that the Y chromosome of Northern European and North American ninespine sticklebacks is much larger than the X chromosome because of a Y chromosome rearrangement, possibly as a result of a duplication of the ancestral Y chromosome or an insertion of a duplicated autosomal segment into the Y chromosome (Ocalewicz *et al.* 2008; Ross *et al.* 2009). However, because the linkage between LG12 and the segment of LG7 was identified both in female and male maps in our study, it is unlikely that the Y chromosome rearrangement is a proximate cause of the rearrangement of linkage patterns. Although it is not certain whether the linkage between LG12 and the segment of LG7 is caused by physical or pseudo linkage, it appears that the segmental region of LG7 co-segregates with the sex-determining locus in Northern European ninespine sticklebacks. Theoretical studies have shown that the formation of linkage between the sex-determining locus and autosomal genes under sexually antagonistic selection has significant consequences on both population fitness and sex chromosome evolution (Charlesworth and Charlesworth 1980; van Doorn and Kirkpatrick 2007). As such, it would be of particular interest to assess if genes underlying sexually dimorphic traits and mating behavior reside in the chromosomal region newly linked to the sex-determining locus.

## Conclusions

Our study demonstrated that the *Pitx1* gene is a strong candidate for the determination of pelvic reduction in Northern European ninespine sticklebacks. The interspecific and intraspecific comparative analyses indicated that although the same genetic mechanism for pelvic reduction might have persisted in threespine and ninespine sticklebacks, alternative QTL for pelvic reduction might have evolved in ninespine sticklebacks, possibly within the past 1.6 million years after the split between Northern European and North American populations. Hence, our study gives empirical support for the view that alternative genetic mechanisms leading to similar phenotypes can evolve over a relatively short evolutionary time scale.

## Supplementary Material

Supporting Information
