# Supplementary material for: Genetic Architecture of Parallel Pelvic Reduction in Ninespine Sticklebacks
Source: G3 (Bethesda). 2013 Oct 1;3(10):1833–42. doi: 10.1534/g3.113.007237 (PMC3789808; doi:10.1534/g3.113.007237)
Supplement: Supporting Information [file supp_3_10_1833__index.html]

Genetic Architecture of Parallel Pelvic Reduction in Ninespine Sticklebacks — Supporting Information 

# Genetic Architecture of Parallel Pelvic Reduction in Ninespine Sticklebacks

## Supporting Information for Shikano *et al.*, 2013

**Files in this Data Supplement:**

- Supporting Information - Figures S1-S2, Files S1-S3, and Tables S1-S4 (PDF, 2 MB)
- Figure S1 - Sex-averaged linkage map of the Northern European ninespine stickleback (PDF, 1 MB)
- Figure S2 - Comparisons of marker order in the Northern European ninespine stickleback with the threespine stickleback (red) and the North American ninespine stickleback (blue). (PDF, 1 MB)
- File S1 - Raw phenotypic data (.txt, 12 KB)
- File S2 - Raw genotyping data (.txt, 499 KB)
- File S3 - Raw map data (.txt, 4 KB)
- Table S1 - Linkage groups and positions of the markers used for linkage maps in the northern European (NE) ninespine stickleback, threespine stickleback genome and North American (NA) ninespine stickleback. (.xlsx, 29 KB)
- Table S2 - Lengths of sex-averaged, female and male maps (.xlsx, 9 KB)
- Table S3 - Average LOD scores (±SD) for pairwise combinations of markers within and between LG7 and LG12 (.xlsx, 8 KB)
- Table S4 - Comparison between phenotypic sex and male-linked alleles at 15 markers on LG12 (.xlsx, 10 KB)
