# Supplementary material for: Genetic Architecture of Parallel Pelvic Reduction in Ninespine Sticklebacks
Source: G3 (Bethesda). 2013 Oct 1;3(10):1833–42. doi: 10.1534/g3.113.007237 (PMC3789808; doi:10.1534/g3.113.007237)
Supplement: Supporting Information [file supp_g3.113.007237_007237SI.pdf]

G3 · Genes | Genomes | Genetics

Genetic Architecture of Parallel Pelvic Reduction in Ninespine Sticklebacks

Takahito Shikano,[*] Veronika N. Laine,[†] Gábor Herczeg,[*,1] Johanna Vilkki,[‡] and Juha Merilä[*]

[*]Ecological Genetics Research Unit, Department of Biosciences, University of Helsinki, FI-00014, Helsinki, Finland
[†]Division of Genetics and Physiology, Department of Biology, University of Turku, FI-20014, Turku, Finland
[‡]MTT Agrifood Research Finland, FI-36100, Jokioinen, Finland

[1]Present address: Behavioural Ecology Group, Department of Systematic Zoology and Ecology, Eötvös Loránd University, Pázmány Péter sétány 1/C, 1117, Hungary

DOI: 10.1534/g3.113.007237

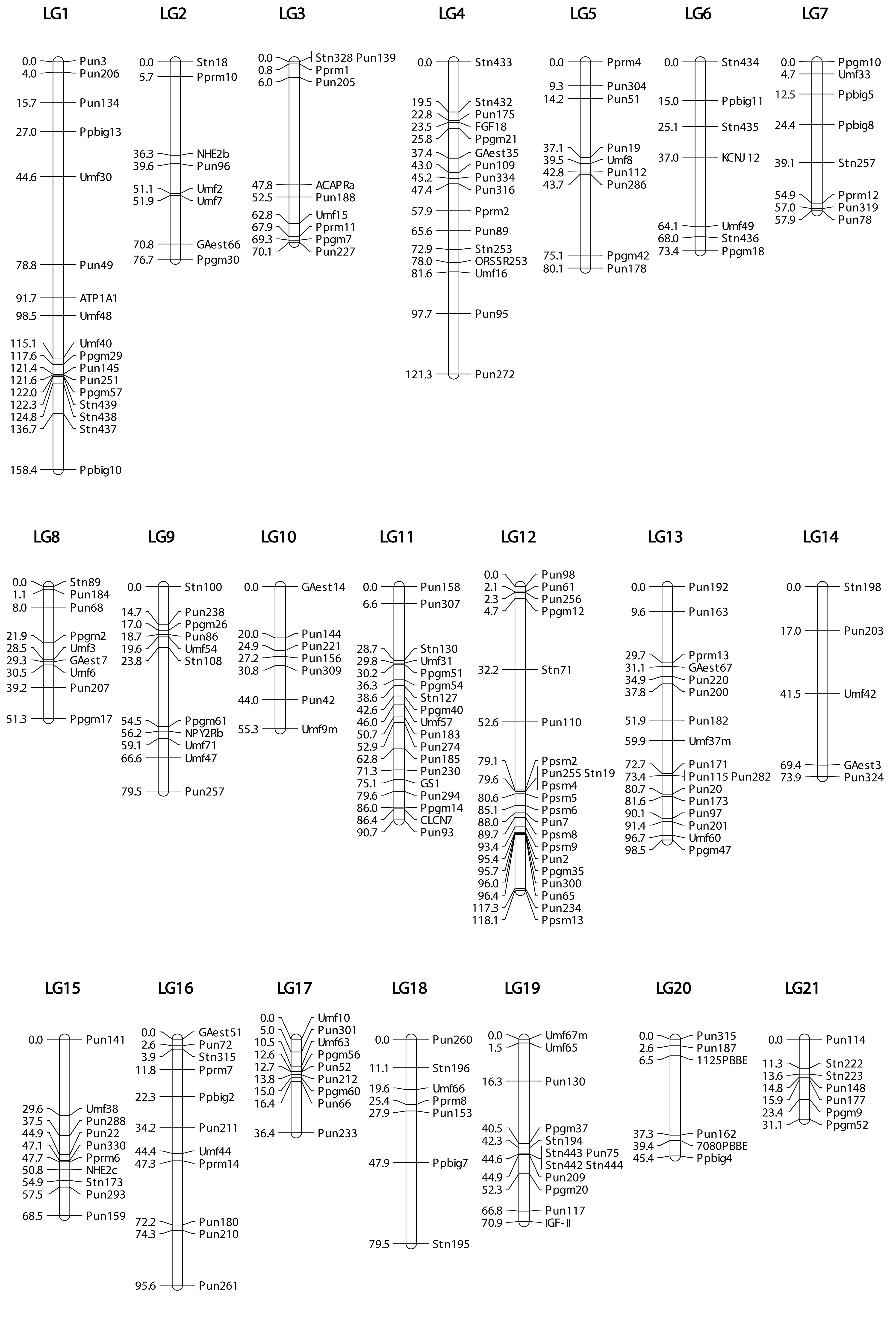

Figure S1 Sex-averaged linkage map of the Northern European ninespine stickleback. Marker positions are given in cM.

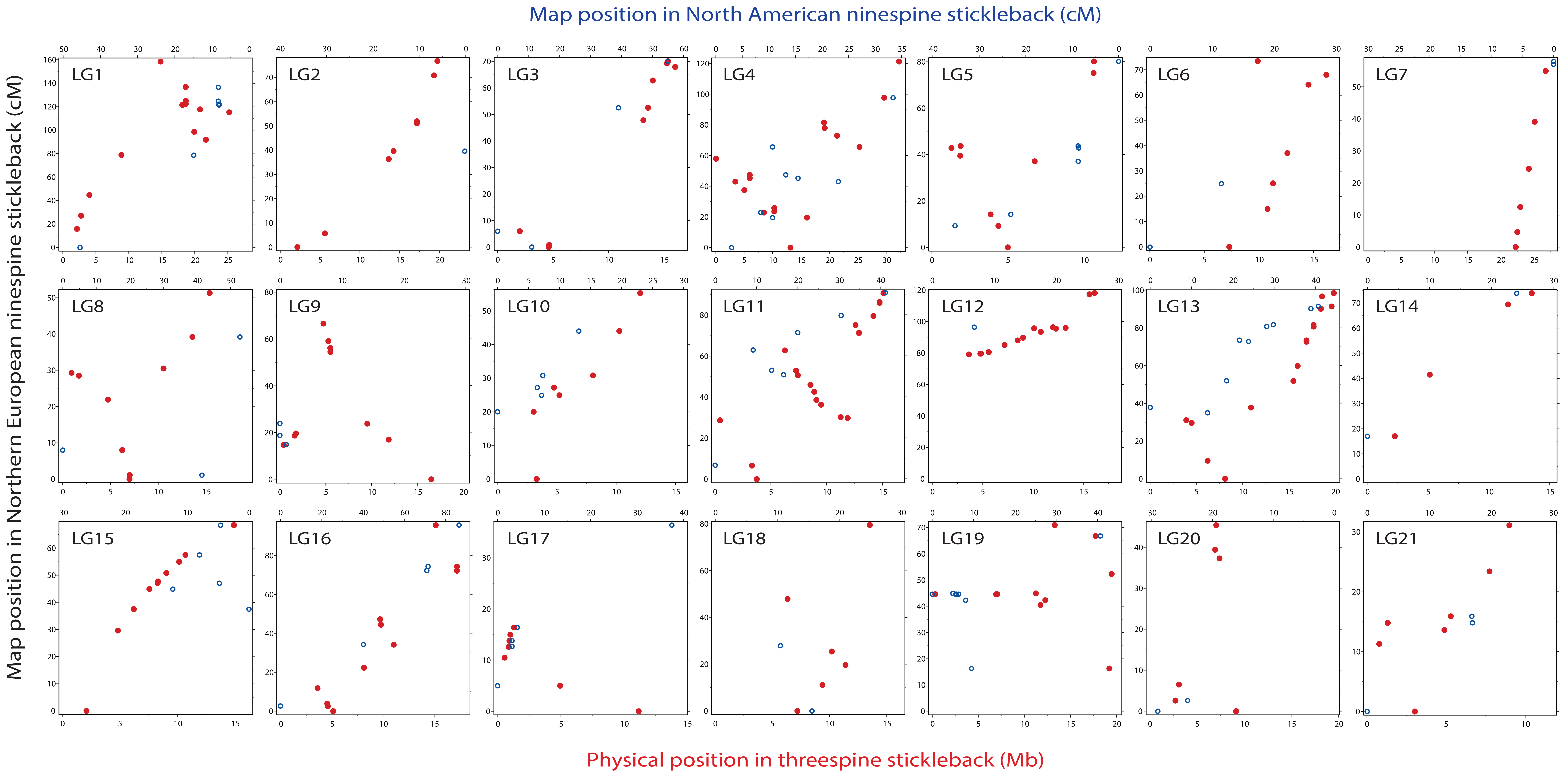

Figure S2 Comparisons of marker order in the Northern European ninespine stickleback with the threespine stickleback (red) and North American ninespine stickleback (blue).

Files S1-S3

Available for download at http://www.g3journal.org/lookup/suppl/doi:10.1534/g3.113.007237/-/DC1

File S1: Raw phenotypic data

File S2: Raw genotyping data

File S3: Raw map data

Tables S1-S4

Available for download at http://www.g3journal.org/lookup/suppl/doi:10.1534/g3.113.007237/-/DC1

Table S1: Linkage groups and positions of the markers used for linkage maps in the northern European (NE) ninespine stickleback, threespine stickleback genome and North American (NA) ninespine stickleback

Table S2: Lengths of sex-averaged, female and male maps

Table S3: Average LOD scores (±SD) for pairwise combinations of markers within and between LG7 and LG12

Table S4: Comparison between phenotypic sex and male-linked alleles at 15 markers on LG12
