# Supplementary material for: Genetic Architecture of Parallel Pelvic Reduction in Ninespine Sticklebacks
Source: G3 (Bethesda). 2013 Oct 1;3(10):1833–42. doi: 10.1534/g3.113.007237 (PMC3789808; doi:10.1534/g3.113.007237)
Supplement: Supporting Information [file supp_g3.113.007237_FigureS2.pdf]

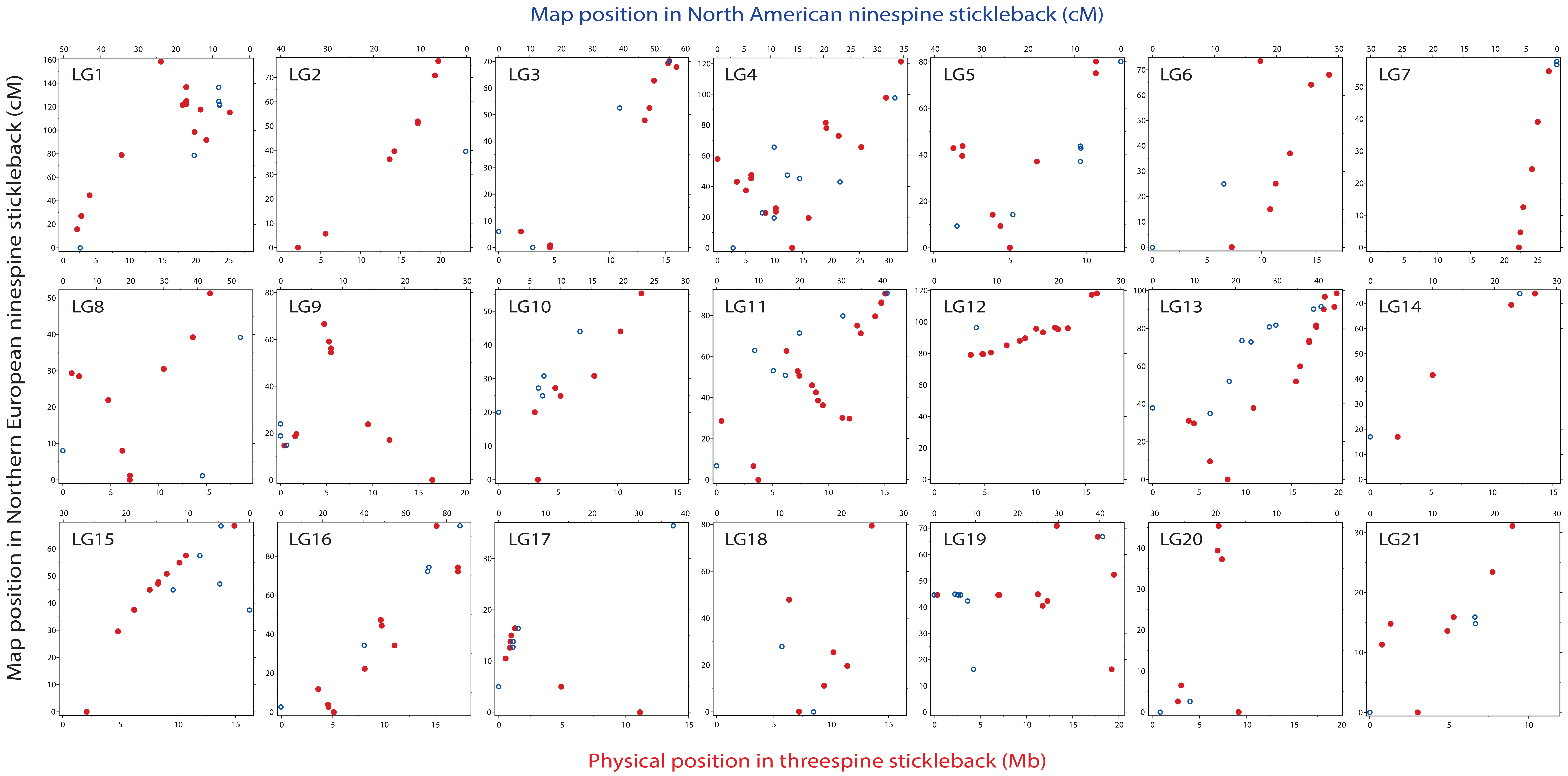

Figure S2 Comparisons of marker order in the Northern European ninespine stickleback with the threespine stickleback (red) and North American ninespine stickleback (blue).
